# 4-(4-Bromo­phen­yl)-4,5,6,7-tetra­hydro-3-methyl-6-oxo-1-phenyl-1*H*-pyrazolo[3,4-*b*]pyridine-5-carbonitrile ethanol solvate

**DOI:** 10.1107/S1600536808028638

**Published:** 2008-09-13

**Authors:** Xue-Sen Fan, Xiao-Yan Li, Xia Wang, Dong-Fang Li, Xin-Ying Zhang

**Affiliations:** a School of Chemistry and Environmental Sciences, Henan Key Laboratory for Environmental Pollution Control, Henan Normal University, Xinxiang, Henan 453007, People’s Republic of China

## Abstract

In the structure of the title compound, C_20_H_15_BrN_4_O·C_2_H_6_O, the hydrogenated pyridinone ring adopts an envelope conformation. The dihedral angle between the bromo-substituted phenyl ring and the pyrazole ring is 79.6 (1)°, and that between the non-substituted phenyl ring and the pyrazole ring is 51.2 (1)°. In the crystal structure, mol­ecules are linked *via* inter­molecular N—H⋯O and O—H⋯N hydrogen bonds. A short inter­molecular N⋯Br contact [3.213 (4) Å] is present in the crystal structure.

## Related literature

For general background, see: Falcó *et al.* (2005 ▶); Kung & Wager (2007 ▶).
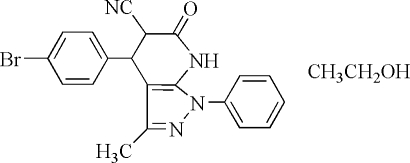


## Experimental

### 

#### Crystal data

C_20_H_15_BrN_4_O·C_2_H_6_O
*M* *_r_* = 453.34
Monoclinic, 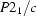

*a* = 21.871 (9) Å
*b* = 9.209 (4) Å
*c* = 10.552 (5) Å
β = 90.370 (5)°
*V* = 2125.4 (15) Å^3^
*Z* = 4
Mo *K*α radiation
μ = 1.96 mm^−1^
*T* = 295 (2) K
0.31 × 0.24 × 0.14 mm

#### Data collection

Bruker SMART CCD area-detector diffractometer
Absorption correction: multi-scan (*SADABS*; Bruker, 1997 ▶) *T*_min_ = 0.586, *T*_max_ = 0.770
10428 measured reflections
3947 independent reflections
2414 reflections with *I* > 2σ(*I*)
*R*_int_ = 0.035

#### Refinement

*R*[*F* ^2^ > 2σ(*F* ^2^)] = 0.046
*wR*(*F* ^2^) = 0.121
*S* = 1.01
3947 reflections
265 parameters
H-atom parameters constrained
Δρ_max_ = 0.53 e Å^−3^
Δρ_min_ = −0.51 e Å^−3^

### 

Data collection: *SMART* (Bruker, 1997 ▶); cell refinement: *SAINT* (Bruker, 1997 ▶); data reduction: *SAINT*; program(s) used to solve structure: *SHELXS97* (Sheldrick, 2008 ▶); program(s) used to refine structure: *SHELXL97* (Sheldrick, 2008 ▶); molecular graphics: *ORTEP-3 for Windows* (Farrugia, 1997 ▶); software used to prepare material for publication: *WinGX* (Farrugia, 1999 ▶).

## Supplementary Material

Crystal structure: contains datablocks I, global. DOI: 10.1107/S1600536808028638/xu2452sup1.cif

Structure factors: contains datablocks I. DOI: 10.1107/S1600536808028638/xu2452Isup2.hkl

Additional supplementary materials:  crystallographic information; 3D view; checkCIF report

## Figures and Tables

**Table 1:** Hydrogen-bond geometry (Å, °)

*D*—H⋯*A*	*D*—H	H⋯*A*	*D*⋯*A*	*D*—H⋯*A*
O2—H2⋯N1^i^	0.82	2.06	2.874 (4)	171
N3—H3*D*⋯O2	0.97	1.84	2.786 (3)	166

Symmetry code: (i) 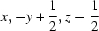.

**Fig. 1.**
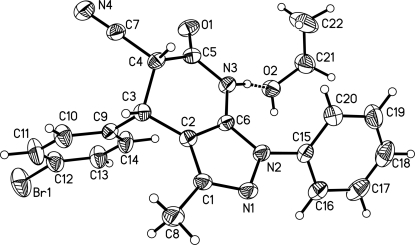
Molecular structure of the title compound with displacement ellipsoids drawn at the 30% probability level. Dashed line indicates hydrogen bonding.

## supplementary crystallographic information

### Comment

Pyrazolo[3,4-*b*]pyridine-6-ones as a subunit of pyrazolo[3,4-*b*]pyridine acted as potential hypnotic drugs in many cases (Falcó *et al.*, 2005). Hydrogenated pyrazolo[3,4-*b*]pyridin-6-ones have been found with good biological activity such as GSK-3 inhibitors (Kung *et al.*, 2007) and have the potential to be used as novel building blocks to construct new nitrogen-containing molecules. The title compound is one of the hydrogenated pyrazolo[3,4-*b*]pyridin-6-one derivatives. Its crystal structure is presented here.

In the title compound (Fig. 1) there are four rings, three planar rings and one nonplanar hydrogenated pyridinone ring. The hydrogenated pyridinone ring is fused to the pyrazole ring and adopts an envelope conformation with C4 at flap position. The dihedral angle between the bromo-substituted benzene ring and the pyrazole ring is 79.6 (1)° and that between the non-substituted phenyl ring and the pyrazole ring is 51.2 (1)°.

Intermolecular N—H···O and O—H···N hydrogen bonding (Table 1) and the weak intermolecular Br1···N4^i^ contact present in the crystal structure [symmetry code: (i) 1-x, -1/2+y, 1/2-z].

### Experimental

4-Bromobenzaldehyde (1 mmol) and ethyl cyanoacetate (1 mmol) were added to 1 ml of 1-butyl-3-methylimidazolium tetrafluoroborate ([bmim][BF_4_]). The mixture was stirred at 353 K until the disappearance of bromobenzaldehyde. Upon cooling to room temperature, 5-amino-3-methyl-1-phenylpyrazole (1 mmol) was added and the mixture was stirred at room temperature for a certain period of time to complete the reaction (monitored by TLC). The reaction time was 9 h totally. Upon completion, the product was not separated from the reaction system; instead, 4 ml of ethanol was added. Single crystals of the title compound were obtained by slow evaporation of the solvent.

### Refinement

H-atoms were included in calculated positions and treated as riding atoms: N—H = 0.97 Å, O—H = 0.82 Å and C—H = 0.93–0.98 Å with *U*_iso_(H) = 1.5U_eq_(CH_3_, OH, NH) and 1.2U_eq_(CH, CH_2_).

### Crystal data

**Table:**

C_20_H_15_BrN_4_O·C_2_H_6_O	*F*(000) = 928
*M**_r_* = 453.34	*D*_x_ = 1.417 Mg m^−^^3^
Monoclinic, *P*2_1_/*c*	Mo *K*α radiation, λ = 0.71073 Å
Hall symbol: -P 2ybc	Cell parameters from 2154 reflections
*a* = 21.871 (9) Å	θ = 2.4–22.3°
*b* = 9.209 (4) Å	µ = 1.96 mm^−^^1^
*c* = 10.552 (5) Å	*T* = 295 K
β = 90.370 (5)°	Block, colourless
*V* = 2125.4 (15) Å^3^	0.31 × 0.24 × 0.14 mm
*Z* = 4	

### Data collection

**Table:**

Bruker SMART CCD area-detector diffractometer	3947 independent reflections
Radiation source: fine-focus sealed tube	2414 reflections with *I* > 2σ(*I*)
graphite	*R*_int_ = 0.035
φ and ω scans	θ_max_ = 25.5°, θ_min_ = 2.4°
Absorption correction: multi-scan (SADABS; Bruker, 1997)	*h* = −26→26
*T*_min_ = 0.586, *T*_max_ = 0.770	*k* = −11→9
10428 measured reflections	*l* = −12→12

### Refinement

**Table:**

Refinement on *F*^2^	Primary atom site location: structure-invariant direct methods
Least-squares matrix: full	Secondary atom site location: difference Fourier map
*R*[*F*^2^ > 2σ(*F*^2^)] = 0.046	Hydrogen site location: inferred from neighbouring sites
*wR*(*F*^2^) = 0.121	H-atom parameters constrained
*S* = 1.01	*w* = 1/[σ^2^(*F*_o_^2^) + (0.0415*P*)^2^ + 1.3425*P*] where *P* = (*F*_o_^2^ + 2*F*_c_^2^)/3
3947 reflections	(Δ/σ)_max_ = 0.001
265 parameters	Δρ_max_ = 0.53 e Å^−^^3^
0 restraints	Δρ_min_ = −0.51 e Å^−^^3^

### Special details

**Table:**

Geometry. All e.s.d.'s (except the e.s.d. in the dihedral angle between two l.s. planes) are estimated using the full covariance matrix. The cell e.s.d.'s are taken into account individually in the estimation of e.s.d.'s in distances, angles and torsion angles; correlations between e.s.d.'s in cell parameters are only used when they are defined by crystal symmetry. An approximate (isotropic) treatment of cell e.s.d.'s is used for estimating e.s.d.'s involving l.s. planes.
Refinement. Refinement of *F*^2^ against ALL reflections. The weighted *R*-factor *wR* and goodness of fit *S* are based on *F*^2^, conventional *R*-factors *R* are based on *F*, with *F* set to zero for negative *F*^2^. The threshold expression of *F*^2^ > σ(*F*^2^) is used only for calculating *R*-factors(gt) *etc*. and is not relevant to the choice of reflections for refinement. *R*-factors based on *F*^2^ are statistically about twice as large as those based on *F*, and *R*- factors based on ALL data will be even larger.

### Fractional atomic coordinates and isotropic or equivalent isotropic displacement parameters (Å^2^)

**Table:**

	*x*	*y*	*z*	*U*_iso_*/*U*_eq_	
Br1	0.449857 (19)	0.58567 (7)	0.18723 (5)	0.1023 (3)	
O1	0.77687 (11)	0.8095 (2)	0.2363 (2)	0.0553 (6)	
O2	0.83439 (11)	0.5202 (3)	0.0508 (2)	0.0558 (6)	
H2	0.8220	0.4430	0.0218	0.084*	
N1	0.77906 (13)	0.2474 (3)	0.4696 (2)	0.0478 (7)	
N2	0.80852 (12)	0.3344 (3)	0.3832 (2)	0.0431 (6)	
N3	0.79796 (12)	0.5800 (3)	0.2978 (2)	0.0434 (6)	
H3D	0.8174	0.5606	0.2174	0.065*	
N4	0.67632 (15)	0.9759 (4)	0.4375 (3)	0.0732 (9)	
C1	0.73512 (15)	0.3303 (4)	0.5195 (3)	0.0442 (8)	
C2	0.73547 (14)	0.4703 (3)	0.4646 (3)	0.0404 (7)	
C3	0.69597 (14)	0.6023 (3)	0.4808 (3)	0.0414 (8)	
H3	0.6870	0.6128	0.5712	0.050*	
C4	0.73741 (14)	0.7328 (3)	0.4401 (3)	0.0407 (8)	
H4	0.7688	0.7432	0.5061	0.049*	
C5	0.77157 (14)	0.7129 (4)	0.3138 (3)	0.0418 (8)	
C6	0.78163 (14)	0.4672 (3)	0.3795 (3)	0.0387 (7)	
C7	0.70350 (16)	0.8703 (4)	0.4370 (3)	0.0487 (8)	
C8	0.69393 (18)	0.2752 (4)	0.6197 (3)	0.0648 (10)	
H8A	0.6546	0.2537	0.5833	0.097*	
H8B	0.6895	0.3477	0.6843	0.097*	
H8C	0.7110	0.1886	0.6561	0.097*	
C9	0.63578 (14)	0.5971 (3)	0.4089 (3)	0.0426 (8)	
C10	0.58214 (16)	0.6426 (4)	0.4648 (3)	0.0628 (10)	
H10	0.5830	0.6750	0.5482	0.075*	
C11	0.52737 (17)	0.6412 (5)	0.3997 (4)	0.0771 (13)	
H11	0.4918	0.6721	0.4391	0.092*	
C12	0.52561 (16)	0.5939 (4)	0.2764 (4)	0.0626 (10)	
C13	0.57776 (16)	0.5502 (4)	0.2179 (3)	0.0661 (11)	
H13	0.5765	0.5189	0.1341	0.079*	
C14	0.63241 (16)	0.5528 (4)	0.2835 (3)	0.0586 (10)	
H14	0.6680	0.5240	0.2427	0.070*	
C15	0.85626 (15)	0.2756 (4)	0.3081 (3)	0.0456 (8)	
C16	0.84675 (18)	0.1459 (4)	0.2460 (3)	0.0559 (9)	
H16	0.8097	0.0969	0.2529	0.067*	
C17	0.8936 (2)	0.0902 (5)	0.1732 (4)	0.0769 (13)	
H17	0.8880	0.0026	0.1309	0.092*	
C18	0.9477 (2)	0.1616 (6)	0.1624 (4)	0.0854 (14)	
H18	0.9786	0.1231	0.1125	0.103*	
C19	0.95680 (19)	0.2899 (6)	0.2249 (4)	0.0816 (13)	
H19	0.9939	0.3384	0.2175	0.098*	
C20	0.91114 (17)	0.3477 (4)	0.2990 (4)	0.0627 (10)	
H20	0.9174	0.4344	0.3423	0.075*	
C21	0.89214 (19)	0.5522 (5)	−0.0008 (4)	0.0750 (12)	
H21A	0.8879	0.5692	−0.0911	0.090*	
H21B	0.9192	0.4699	0.0114	0.090*	
C22	0.9183 (3)	0.6785 (6)	0.0587 (6)	0.128 (2)	
H22A	0.8972	0.7637	0.0298	0.192*	
H22B	0.9607	0.6855	0.0371	0.192*	
H22C	0.9144	0.6704	0.1490	0.192*	

### Atomic displacement parameters (Å^2^)

**Table:**

	*U*^11^	*U*^22^	*U*^33^	*U*^12^	*U*^13^	*U*^23^
Br1	0.0484 (3)	0.1630 (6)	0.0952 (4)	0.0022 (3)	−0.0134 (2)	−0.0219 (3)
O1	0.0811 (17)	0.0401 (14)	0.0446 (13)	−0.0010 (13)	0.0004 (11)	0.0073 (11)
O2	0.0628 (16)	0.0541 (16)	0.0508 (13)	−0.0062 (13)	0.0098 (11)	−0.0147 (12)
N1	0.0548 (17)	0.0385 (16)	0.0503 (16)	−0.0019 (14)	0.0086 (13)	0.0083 (13)
N2	0.0485 (16)	0.0365 (16)	0.0444 (14)	0.0022 (14)	0.0089 (12)	0.0029 (13)
N3	0.0540 (16)	0.0375 (16)	0.0387 (14)	0.0027 (13)	0.0093 (12)	0.0032 (12)
N4	0.065 (2)	0.049 (2)	0.105 (3)	0.0064 (18)	−0.0053 (18)	−0.0055 (19)
C1	0.0478 (19)	0.039 (2)	0.0458 (18)	−0.0049 (17)	0.0068 (15)	0.0041 (16)
C2	0.0456 (18)	0.039 (2)	0.0366 (16)	0.0001 (16)	0.0007 (14)	0.0015 (14)
C3	0.0476 (18)	0.042 (2)	0.0344 (15)	−0.0009 (16)	0.0043 (13)	−0.0030 (14)
C4	0.0491 (18)	0.0367 (19)	0.0364 (16)	0.0024 (16)	−0.0051 (14)	−0.0046 (14)
C5	0.0486 (19)	0.040 (2)	0.0371 (16)	−0.0037 (16)	−0.0039 (14)	0.0000 (16)
C6	0.0447 (18)	0.0332 (19)	0.0383 (16)	0.0012 (15)	0.0017 (14)	0.0015 (14)
C7	0.052 (2)	0.040 (2)	0.054 (2)	−0.0029 (18)	−0.0030 (16)	−0.0052 (16)
C8	0.074 (3)	0.053 (2)	0.068 (2)	−0.001 (2)	0.024 (2)	0.0119 (19)
C9	0.0445 (18)	0.0367 (19)	0.0466 (18)	0.0004 (15)	0.0051 (14)	−0.0050 (15)
C10	0.055 (2)	0.084 (3)	0.049 (2)	−0.001 (2)	0.0105 (17)	−0.0201 (19)
C11	0.043 (2)	0.117 (4)	0.072 (3)	0.004 (2)	0.0126 (19)	−0.022 (3)
C12	0.043 (2)	0.075 (3)	0.069 (2)	−0.001 (2)	−0.0050 (18)	−0.009 (2)
C13	0.054 (2)	0.087 (3)	0.057 (2)	0.005 (2)	−0.0018 (18)	−0.025 (2)
C14	0.047 (2)	0.073 (3)	0.056 (2)	0.0102 (19)	0.0020 (16)	−0.0236 (19)
C15	0.050 (2)	0.043 (2)	0.0433 (17)	0.0105 (17)	0.0019 (15)	0.0024 (16)
C16	0.071 (2)	0.046 (2)	0.051 (2)	0.0061 (19)	0.0051 (18)	−0.0011 (17)
C17	0.119 (4)	0.052 (3)	0.060 (2)	0.021 (3)	0.017 (2)	−0.004 (2)
C18	0.091 (4)	0.086 (4)	0.080 (3)	0.038 (3)	0.034 (3)	0.012 (3)
C19	0.054 (3)	0.093 (4)	0.098 (3)	0.008 (3)	0.017 (2)	0.007 (3)
C20	0.054 (2)	0.062 (3)	0.072 (2)	0.002 (2)	0.0058 (19)	−0.001 (2)
C21	0.071 (3)	0.079 (3)	0.075 (3)	−0.010 (2)	0.016 (2)	−0.015 (2)
C22	0.105 (4)	0.127 (5)	0.152 (5)	−0.062 (4)	0.036 (4)	−0.049 (4)

### Geometric parameters (Å, °)

**Table:**

Br1—C12	1.901 (4)	C9—C14	1.386 (4)
O1—C5	1.215 (3)	C10—C11	1.377 (5)
O2—C21	1.409 (4)	C10—H10	0.9300
O2—H2	0.8200	C11—C12	1.373 (5)
N1—C1	1.338 (4)	C11—H11	0.9300
N1—N2	1.377 (3)	C12—C13	1.361 (5)
N2—C6	1.357 (4)	C13—C14	1.378 (5)
N2—C15	1.422 (4)	C13—H13	0.9300
N3—C5	1.364 (4)	C14—H14	0.9300
N3—C6	1.398 (4)	C15—C20	1.376 (5)
N3—H3D	0.9687	C15—C16	1.377 (5)
N4—C7	1.140 (4)	C16—C17	1.383 (5)
C1—C2	1.414 (4)	C16—H16	0.9300
C1—C8	1.482 (4)	C17—C18	1.359 (6)
C2—C6	1.356 (4)	C17—H17	0.9300
C2—C3	1.502 (4)	C18—C19	1.367 (6)
C3—C9	1.516 (4)	C18—H18	0.9300
C3—C4	1.567 (4)	C19—C20	1.379 (5)
C3—H3	0.9800	C19—H19	0.9300
C4—C7	1.468 (5)	C20—H20	0.9300
C4—C5	1.544 (4)	C21—C22	1.438 (6)
C4—H4	0.9800	C21—H21A	0.9700
C8—H8A	0.9600	C21—H21B	0.9700
C8—H8B	0.9600	C22—H22A	0.9600
C8—H8C	0.9600	C22—H22B	0.9600
C9—C10	1.381 (5)	C22—H22C	0.9600
			
C21—O2—H2	109.5	C9—C10—H10	119.2
C1—N1—N2	105.7 (2)	C12—C11—C10	119.6 (3)
C6—N2—N1	109.8 (2)	C12—C11—H11	120.2
C6—N2—C15	130.3 (3)	C10—C11—H11	120.2
N1—N2—C15	119.7 (3)	C13—C12—C11	120.4 (3)
C5—N3—C6	118.8 (3)	C13—C12—Br1	119.6 (3)
C5—N3—H3D	117.5	C11—C12—Br1	120.0 (3)
C6—N3—H3D	121.2	C12—C13—C14	119.6 (3)
N1—C1—C2	110.6 (3)	C12—C13—H13	120.2
N1—C1—C8	121.8 (3)	C14—C13—H13	120.2
C2—C1—C8	127.5 (3)	C13—C14—C9	121.7 (3)
C6—C2—C1	105.0 (3)	C13—C14—H14	119.1
C6—C2—C3	121.6 (3)	C9—C14—H14	119.1
C1—C2—C3	133.4 (3)	C20—C15—C16	121.0 (3)
C2—C3—C9	114.6 (3)	C20—C15—N2	120.0 (3)
C2—C3—C4	104.8 (2)	C16—C15—N2	119.1 (3)
C9—C3—C4	112.9 (2)	C15—C16—C17	118.4 (4)
C2—C3—H3	108.1	C15—C16—H16	120.8
C9—C3—H3	108.1	C17—C16—H16	120.8
C4—C3—H3	108.1	C18—C17—C16	121.0 (4)
C7—C4—C5	109.3 (3)	C18—C17—H17	119.5
C7—C4—C3	112.0 (3)	C16—C17—H17	119.5
C5—C4—C3	115.4 (2)	C17—C18—C19	120.1 (4)
C7—C4—H4	106.5	C17—C18—H18	119.9
C5—C4—H4	106.5	C19—C18—H18	119.9
C3—C4—H4	106.5	C18—C19—C20	120.2 (4)
O1—C5—N3	122.1 (3)	C18—C19—H19	119.9
O1—C5—C4	122.9 (3)	C20—C19—H19	119.9
N3—C5—C4	114.9 (3)	C15—C20—C19	119.3 (4)
C2—C6—N2	108.9 (3)	C15—C20—H20	120.4
C2—C6—N3	125.9 (3)	C19—C20—H20	120.4
N2—C6—N3	125.1 (3)	O2—C21—C22	110.8 (3)
N4—C7—C4	178.1 (4)	O2—C21—H21A	109.5
C1—C8—H8A	109.5	C22—C21—H21A	109.5
C1—C8—H8B	109.5	O2—C21—H21B	109.5
H8A—C8—H8B	109.5	C22—C21—H21B	109.5
C1—C8—H8C	109.5	H21A—C21—H21B	108.1
H8A—C8—H8C	109.5	C21—C22—H22A	109.5
H8B—C8—H8C	109.5	C21—C22—H22B	109.5
C10—C9—C14	117.1 (3)	H22A—C22—H22B	109.5
C10—C9—C3	121.0 (3)	C21—C22—H22C	109.5
C14—C9—C3	121.8 (3)	H22A—C22—H22C	109.5
C11—C10—C9	121.6 (3)	H22B—C22—H22C	109.5
C11—C10—H10	119.2		
			
C1—N1—N2—C6	−1.3 (3)	C5—N3—C6—C2	9.3 (4)
C1—N1—N2—C15	−176.8 (3)	C5—N3—C6—N2	−172.9 (3)
N2—N1—C1—C2	0.7 (3)	C5—C4—C7—N4	168 (11)
N2—N1—C1—C8	−178.6 (3)	C3—C4—C7—N4	39 (12)
N1—C1—C2—C6	0.0 (3)	C2—C3—C9—C10	137.1 (3)
C8—C1—C2—C6	179.4 (3)	C4—C3—C9—C10	−103.1 (4)
N1—C1—C2—C3	177.5 (3)	C2—C3—C9—C14	−45.7 (4)
C8—C1—C2—C3	−3.1 (6)	C4—C3—C9—C14	74.2 (4)
C6—C2—C3—C9	97.3 (3)	C14—C9—C10—C11	1.3 (6)
C1—C2—C3—C9	−79.9 (4)	C3—C9—C10—C11	178.7 (4)
C6—C2—C3—C4	−27.0 (4)	C9—C10—C11—C12	−0.1 (7)
C1—C2—C3—C4	155.8 (3)	C10—C11—C12—C13	−0.8 (7)
C2—C3—C4—C7	173.7 (2)	C10—C11—C12—Br1	178.0 (3)
C9—C3—C4—C7	48.4 (3)	C11—C12—C13—C14	0.5 (6)
C2—C3—C4—C5	47.8 (3)	Br1—C12—C13—C14	−178.4 (3)
C9—C3—C4—C5	−77.5 (3)	C12—C13—C14—C9	0.8 (6)
C6—N3—C5—O1	−169.4 (3)	C10—C9—C14—C13	−1.7 (6)
C6—N3—C5—C4	13.4 (4)	C3—C9—C14—C13	−179.0 (3)
C7—C4—C5—O1	11.5 (4)	C6—N2—C15—C20	54.7 (5)
C3—C4—C5—O1	138.8 (3)	N1—N2—C15—C20	−130.8 (3)
C7—C4—C5—N3	−171.3 (3)	C6—N2—C15—C16	−125.7 (4)
C3—C4—C5—N3	−44.0 (4)	N1—N2—C15—C16	48.7 (4)
C1—C2—C6—N2	−0.8 (3)	C20—C15—C16—C17	−0.6 (5)
C3—C2—C6—N2	−178.7 (3)	N2—C15—C16—C17	179.9 (3)
C1—C2—C6—N3	177.2 (3)	C15—C16—C17—C18	−0.2 (6)
C3—C2—C6—N3	−0.6 (5)	C16—C17—C18—C19	0.6 (7)
N1—N2—C6—C2	1.3 (3)	C17—C18—C19—C20	−0.1 (7)
C15—N2—C6—C2	176.2 (3)	C16—C15—C20—C19	1.0 (5)
N1—N2—C6—N3	−176.7 (3)	N2—C15—C20—C19	−179.5 (3)
C15—N2—C6—N3	−1.8 (5)	C18—C19—C20—C15	−0.7 (6)

### Hydrogen-bond geometry (Å, °)

**Table:**

*D*—H···*A*	*D*—H	H···*A*	*D*···*A*	*D*—H···*A*
O2—H2···N1^i^	0.82	2.06	2.874 (4)	171.
N3—H3D···O2	0.97	1.84	2.786 (3)	166.

Symmetry codes: (i) *x*, −*y*+1/2, *z*−1/2.
